# The Operative Treatment of Cancer of the Breast

**Published:** 1898-12-10

**Authors:** 


### THE OPERATIVE TREATMENT OF CANCER OF THE BREAST.

In a recently delivered clinical lecture on Halsted's operation for the removal of cancer of the breast, Mr. Butlin speaks very highly of that proceeding, which, since the year 1895, he has made his routine proceeding. It should be noted that the researches of Heidenhain and Stiles have shown that cancer of the breast spreads rapidly backwards towards, and then into, the pectoral fascia, even when there appears to the naked eye to be a considerable interval between the posterior border of the tumour and the fascia. Moreover, there is considerable difficulty in satisfactorily separating the fascia from the muscle, and both the investigators agree in believing that the cancer cells are so soon and so widely conveyed by the lymphatics of the breast, and especially towards the pectoral fascia, that the disease is not entirely removed by the operations usually performed. Although they differ in certain details as to the direction of distribution by the lymphatics, they both agree that the whole breast should be removed. It appeared, then, to Mr. Butlin that an operation designed to remove thoroughly all the structures in which the cancer of the breast may be disseminated would be the means of saving the lives of, at least, some women affected by the disease, and that of the various operations which had been proposed the one designed and practised by Halsted best fulfilled the requirements.

In this operation everything which is removed comes away in one continuous mass, and this mass contains the whole of the mammary gland, the whole of the pectoral muscle, with, perhaps, the exception of the clavicular portion, the fascia beneath the pectoral muscle and that in front of and behind the lesser pectoral, the loose connective tissue on the side and back walls of the axilla, the fat and all which it contains right up to the clavicle. This operation is, of course, a serious one, but if care is taken to lessen the hæmorrhage by operating deliberately and taking up every vessel as soon as, or even before it bleeds, it is not more dangerous than any other large operation for the same disease. Between the beginning of 1895 and the end of 1897 Mr. Butlin has performed it 33 times with one death.

Now as to results. Taking those that have been done three years and over, there are 13 cases only to consider, and, of these, nine were alive and well when last seen or heard of (from their medical men) at periods of from three to four years after the operation. In more than one of the successful cases the glands in the axilla were cancerous.

In none of the cases did he remove the supraclavicular glands, for he is inclined to agree with Cheyne in thinking that cases in which these are affected are hopeless. But on that point he reserves his opinion. Mr. Butlin says that he is not so sanguine as to believe that surgeons are going to cure every case of cancer of the breast at some not far distant time, but he does believe that far better results will be secured in the future than we have been accustomed to in the past and if the results given by Mr. Watson Cheyne and by Professor Halsted are added to those which he now describes, it will be found that out of 42 patients treated by the three operators, more than three years ago, 23 are still alive and well, or have died of some other cause than cancer after passing the three years' limit. "And these results have been secured under the most disheartening circumstances. There is hardly a single case in which the disease was not known to have existed for several months, and in many of them it had been noted for one, two, or more years. There were several of them in which the primary tumour was ulcerated, and several in which the axillary glands were filled with cancer."

We think that these results should be taken note of, for if once the general practitioner gets rid of the pessimism with which the whole subject was overlain a few years ago, and, indeed, still is in the minds of many men, cases will be sent up for operation at a far earlier date than has hitherto been the case, and the results may thus become proportionally better.

